# Supplementary material for: School health assessment tools: a systematic review of measurement in primary schools
Source: PeerJ. 2020 Jul 21;8:e9459. doi: 10.7717/peerj.9459 (PMC7380271; doi:10.7717/peerj.9459)
Supplement: Supplemental Information 1 [file peerj-08-9459-s001.docx]

**School health assessment tools: a systematic review of measurement in primary schools**

Maryam Kazemitabar ^a^, Ali Moghadamzadeh ^b^, Mojtaba Habibi ^c,d^, Rezvan Hakimzadeh ^e^, Danilo Garcia ^f,g,h^

^a^Department of Curriculum and Instruction, Faculty of Psychology and Educational Sciences, University of Tehran, Tehran, Iran

^b^Department of Curriculum and Instruction, Faculty of Psychology and Educational Sciences, University of Tehran, Tehran, Iran

^c^Health Promotion Research Center, Iran University of Medical Sciences, Tehran, Iran

^d^Department of Health Psychology, School of Behavioral Sciences and Mental Health (Tehran Institute of Psychiatry), Iran University of Medical Sciences, Tehran, Iran

^e^Department of Curriculum and Instruction, Faculty of Psychology and Educational Sciences, University of Tehran, Tehran, Iran

^f^Blekinge Center of Competence, Region Blekinge, Karlskrona, Sweden

^g^Department of Behavioral Sciences and Learning, Linköping University, Linköping, Sweden

^h^Department of Psychology, University of Gothenburg, Gothenburg, Sweden

**RATIONALE**

To the best of our knowledge, there are no other systematic reviews that investigate the measurement properties of school health assessment tools. This systematic review will help researchers and practitioners to choose the proper tool for school health assessment based on the tool’s psychometric properties. Moreover, by pointing out methodological issues, this systematic review will help researchers and practitioners involved in test development and test validation to recognize and consider how these issues influence their studies. Finally, we implement the COSMIN Risk of Bias checklist as a manual for tool development and psychometric studies that guide researchers to select suitable statistical methods to evaluate measurement properties of the assessment tool at hand.

**CONTRIBUTION**

While the number of studies included in this review is relatively small, our findings suggest that there are just a few tools measuring school health. That is, the implementation of the COSMIN Risk Bias checklist allowed us to point out the lack of psychometric studies addressing school health measures. More specifically, there is a gap of studies assessing measurement error and construct validity. In addition, we also found that most of the studies included in the systematic review reported Cronbach’s alpha as a reliability measure. This is not correct, since alpha is a measure for internal consistency, while for reliability a test-retest measure is needed. In addition, some of the studies reported EFA and CFA results as tests of construct validity, despite the fact that these types of analyses test structural validity. For evaluating construct validity, researchers need tools that compare convergent or discriminate validity/known group validity. On this basis, we have recommended the consideration of these shortcomings and the implementation of the COSMIN Risk Bias checklist for tool development and psychometric studies.
