# Supplementary material for: School health assessment tools: a systematic review of measurement in primary schools
Source: PeerJ. 2020 Jul 21;8:e9459. doi: 10.7717/peerj.9459 (PMC7380271; doi:10.7717/peerj.9459)
Supplement: Supplemental Information 2 [file peerj-08-9459-s002.doc]

| **Section/topic** | **#** | **Checklist item** | **Reported on page #** |
| --- | --- | --- | --- |
| **TITLE** | | |  |
| Title | 1 | **School Health Assessment Tools: A Systematic Review of Measurement in Primary Schools** | 1 |
| **ABSTRACT** | | |  |
| Structured summary | 2 | **Abstract**  ***Background.*** This systematic review investigates the psychometric properties of the school health’s assessment tools in primary schools through COSMIN Risk of Bias checklist. The authors examined the studies that measures the measurement properties of school-health instruments to give a clear overview of quality of all available tools measuring school health in primary schools.  ***Method.*** Databases of EBSCOhost, PubMed, ProQuest, Wily, PROSPERO, and OpenGrey were systematically searched without any time limitation to find all full-text English journal articles that studied at least one of the COSMIN checklist measurement properties of a school-health assessment tool in primary schools. The instruments should be constructed based on WHO’s Health-Promoting Schools framework. The eligible studies were assessed by COSMIN Risk of Bias checklist to report their quality of methodology for each measurement property and for the whole study by rating high, medium or poor quality.  ***Results.*** At the final screening just seven studies remained to review. Four studies were tool development, three of them rated “adequate” and the other study received “very good”; five studies examined the content validity, four of them assessed as “very good”, the remaining one study rated as “adequate”. All seven studies measured structural validity, three of them rated as “very good”, one of them got an “adequate” rating and the other three studies received “inadequate”. All the seven studies investigated the internal consistency, five of them assessed as “very good”, one rated as “doubtful’ and the last one “inadequate”. Just two studies examined the cross-cultural validity and both studies received a “doubtful” rating quality. Finally, all seven studies measured reliability, two of them rated as “very good” and the rest five studies assessed as “doubtful” based on COSMIN checklist criteria for quality of measurement properties assessment.  ***Conclusion.*** Based on the information drawn from the studies, the number of studies assessed school health assessment tools is not sufficient and there is a serious need to investigate the psychometric properties of the available instruments measuring school health at primary schools. Moreover, the conducted studies did not fulfilled all the criteria of the COSMIN checklist for assessing measurement properties. The present systematic review suggests future studies consider these criteria for measuring psychometric properties and developing school health assessment tools. | 3 |
| **INTRODUCTION** | | |  |
| Rationale | 3 | Kolbe and Allensworth (1987) presented eight components of the Coordinated School Health Program to promote health at schools. These components are: health education (1), physical education (2), health services (3), nutrition services (4), counseling, psychological and social services (5), healthy environment (6), school-site health promotion for staff (7), and family and community involvement (8) [15]. Furthermore, the World Health Organization developed the Health Promoting Schools framework in 1996 [16], which introduced components to promote health at schools. This framework includes three main characteristics of School Curriculum, Ethos and/or Environment, and Families and/or Communities [17] which are divided into six key Health-Promoting School factors of healthy school policies, physical school environment, social school environment, health skills and education, links with parents and community, and access to health services at the school. Later the Whole School, Whole Community, Whole Child model was released in 2014 and includes 11 components of health services, health education, employee wellness, counseling, psychological and social services, nutrition environment and services, physical education and physical activity, physical environment, social emotional climate, family engagement, and community involvement [18]. This model endeavors to link health and educational outcomes and is a combination of the Coordinated School Health model and the Association for Supervision and Curriculum Development’s Whole Child approach. Furthermore, the National Association of School Nurses introduced in 2015 the Framework for 21th Century School Nursing Practice, a student-centered nursing care approach that connects students, family and school community. This framework consists of 5 components including standards of practice (1), care coordination (2), leadership (3), quality improvement (4), and community/public health (5) [19]. As mentioned, there are different models for school health concept, but this review assesses the instruments that are developed to measure WHO’s Health-Promoting Schools (HPS) components. | 4-5 |
| Objectives | 4 | This systematic review seeks to (1) identify existing instruments that measure the components of school health at primary schools based on WHO Health-Promoting Schools Framework, and (2) evaluate these measures’ psychometric properties. All studies have been screened for the risk of bias and quality using the modified COnsensus-based Standards for the selection of health Measurement INstruments (COSMIN) checklist. This review will help to clarify if the current instruments, tools, scales, and indexes measure the components | 5 |
| **METHODS** | | |  |
| Protocol and registration | 5 | The review protocol of the study used the Preferred Reporting Items for Systematic Review and Meta-Analyses (PRISMA) guidelines. This review was registered with the International Prospective Registry of Systematic Reviews (PROSPERO) (#158158)[[1]](#footnote-2) before starting the research according to the PRISMA guideline recommendation [42]. | 5 |
| Eligibility criteria | 6 | The criteria for the selection of the studies included in this systematic review were those that: (1) developed an instrument(s) to measure school health; (2) the instruments should measure the WHO health-promoting components (at least four of the eight components); (3) evaluated at least one of the psychometric properties described in COSMIN Risk of Bias checklist for a school health instrument; (4) the instrument(s) was constructed for primary school children; (5) were original research journal articles (i.e., book chapters, thesis and case studies were excluded); and (6) were in English. All articles for which full texts were not available online were also excluded from the study. | 5 |
| Information sources | 7 | The studies were searched through the EBSCO(host), ProQuest, PubMed, and Wily databases, also a gray literature search was performed in PROSPERO, OpenGrey, and Google Scholar. No publication date was considered. Each database was queried from November 2019 using the Boolean operators (AND/OR). The terms searched were: “*school health”, “health-promoting schools”, “coordinated school health”, “psychometric properties”, “reliability”, “validity”, “tool”, “scale”, “index”, “instrument”,* and *“questionnaire”*. We also conducted hand search on the references lists of the selected studies. | 6 |
| Search | 8 | The search algorithm in EBSCOhost was: TI ( (“school health”) AND (scale OR index OR tool OR instrument OR questionnaire OR evaluation OR “psychometric properties” OR reliability OR validity) ) AND AB ( (“school health”) AND (scale OR index OR tool OR instrument OR questionnaire OR evaluation OR “psychometric properties” OR reliability OR validity) ) and the PubMed search algorithm was: ((((“school health”)[Title] AND (scale[Title] OR index[Title] OR tool[Title] OR instrument[Title] OR questionnaire[Title] OR evaluation[Title] OR “psychometric properties”[Title] OR reliability[Title] OR validity)[Title])) AND ((“school health”) AND (scale OR index OR tool OR instrument OR questionnaire OR evaluation OR “psychometric properties” OR reliability OR validity)[MeSH Terms])) AND ((“school health”) AND (scale OR index OR tool OR instrument OR questionnaire OR evaluation OR “psychometric properties” OR reliability OR validity)). | 6 |
| Study selection | 9 | We identified 649 studies for this review. Three authors examined the studies and screening them by titles and abstracts in terms of relatedness, then the full-texts of the selected articles were studied. In case of any discrepancy among raters MK and MH, the case was discussed until consensus concerning eligibility was reached. Articles related to school health assessment but that did not report the psychometric properties of the used instrument [50-53] or that included middle or high school students [54-56] were excluded from this systematic review. Finally, seven articles were selected as clearly relevant for inclusion in the systematic review (Figure 1) | 6 |
| Data collection process | 10 | The first reviewer (MK) developed a customized data extraction form, and extracted characteristics data of the studies and participants; the second reviewer (MH) also extracted the data independently, the results of the two extractions were compared and in case of any differences the third reviewer (AM) intervened to reach consensus. | 7 |
| Data items | 11 | The data regarding characteristics of the selected studies included: first author name, year of publication, country, sample size, time to answer the items, number of items, response scale, participant age/grade, participant sex, and instrument factors (Table 1). | 7 |
| Risk of bias in individual studies | 12 | The selected studies (*n* = 7) were assessed by the modified COSMIN checklist, which was developed to assess the methodological quality of studies on measurement properties. The COSMIN checklist contains 10 parameters including the following psychometric properties: PROM development, content validity, structural validity, internal consistency, cross-cultural validity/measurement invariance, reliability, measurement errors, and hypotheses testing for construct validity. The option “not applicable” is included and applies for studies that did not investigated a specific psychometric property and were therefore not included. Each parameter’s check box has items to assess each specific psychometric property using a four-point scale (“*very good*”, “*adequate*”, “*doubtful*”, and “*inadequate*”). The overall quality of each psychometric property is operationalized as the lowest rating of any standard in the box is taken (i.e. “the worst score counts” principle) [57-59]. In this study each “very good” rating took 1 score and the options “adequate”, “doubtful”, and “inadequate” took 0. That is, the maximum score for a study was 8. The final quality of any individual study was rated based on high quality for any study take 50% of scores or more, moderate quality for 30% to 50%, and low quality for below 30% of scores. The modified version of the COSMIN checklist was used in this review; in the modified version two measurement properties of *“criterion validity”* and *“responsiveness”* is omitted because none of the studies were investigated these properties. | 6-7 |
| Summary measures | 13 | No meta-analysis |  |
| Synthesis of results | 14 | No meta-analysis |  |

Page 1 of 2

| **Section/topic** | **#** | **Checklist item** | **Reported on page #** |
| --- | --- | --- | --- |
| Risk of bias across studies | 15 | No meta-analysis |  |
| Additional analyses | 16 | No meta-analysis |  |
| **RESULTS** | | |  |
| Study selection | 17 | The search strategy performed through 6 data bases of EBSCOhost, PubMed, ProQuest, Wily, PROSPERO, and OpenGrey that resulted in 952 article; after removing the duplicates 649 articles remained, after screening of title and abstracts 515 articles screened; then the full-text of the articles were studied for investigating the eligibility criteria including original journal articles, articles that studies at least one of the COSMIN Risk of Bias checklist measure properties of a school health assessment tool, study’s population should be primary schools, articles of English language and articles with full-text availability. Finally, seven articles remained for inclusion in this systematic review (Figure 1). | 7 |
| Study characteristics | 18 | Descriptive characteristics of the included articles were extracted which are first author name, publication year, country, sample size, time to answer the items/number of items, response scale (multiple/forced choice, Likert scale, fill-in-the blank, true or false, yes or no, and text response), participant age/grade, participant sex, instrument’s factors (Table 1). | 8 |
| Risk of bias within studies | 19 | Table 2 shows the modified COSMIN risk of bias checklist for the studies included in this systematic review. In the modified version of the checklist “criterion validity” and “responsiveness” were omitted because none of the instruments reported these items. The term “tool development” was substituted for the term “Patient Reported Outcome Measure (PROM) development” in order to reflect the real target population of the studies which are students, teachers, and principals instead of patient. | 8 |
| Results of individual studies | 20 | The articles were assessed by the three raters and all of the raters were agreed that none of the seven articles were scored “high quality”, four of them assessed as “moderate quality”, and the rest three articles as “low quality”. | 8-10 |
| Synthesis of results | 21 | No meta-analysis |  |
| Risk of bias across studies | 22 | No meta-analysis |  |
| Additional analysis | 23 | No meta-analysis |  |
| **DISCUSSION** | | |  |
| Summary of evidence | 24 | This systematic review have assessed the measurement properties of school health assessment tools used in primary schools. The instruments selected followed the WHO’s Health-Promoting Schools framework. Despite the importance of existing valid and reliable tools to measure school health, the number of school health assessment tools were few. Moreover, for instruments like CDC’s School Health Index and CDC’s School Health Profile, our search strategy was not able to find any study measuring their psychometric properties. Nevertheless, we systematically found seven studies that were recognized as eligible for our criteria. As far as we know, this is the first systematic review that has methodologically studied the quality of school health assessment tools. | 10-11 |
| Limitations | 25 | In sum, we found only a few instruments that assess school health. What is more, there were quite a few studies measuring the psychometric properties of these instruments. Importantly, this small number of studies did not consider some of the measurement properties mentioned in the COSMIN checklist. Thus, there is not enough evidence on the quality of these instruments. This systematic review suggests that researchers need to conduct more studies on school health assessment tools in order to acquire better conclusions on these instrument’s measurement properties. | 11 |
| Conclusions | 26 | The studies included in this systematic review suggested that the instruments used in this studies had a relatively strong internal consistency and content validity, a moderate quality for structural validity and tool development and poor quality for reliability, cross-cultural validity, measurement error, and hypotheses testing for construct validity. Due to the inconsistency of school health assessment tools, we recommended that future studies on measurement properties of school health assessment tools adhere to the measurement properties of the COSMIN Risk of Bias checklist in order to estimate a more comprehensive and complete report for psychometric properties of the school health instruments. | 11 |
| **FUNDING** | | |  |
| Funding | 27 | This work was supported by a grant from The Swedish Research Council (Dnr. 2015-01229). The funders had no role in study design, data collection and analysis, decision to publish, or preparation of the manuscript. |  |

*From:*  Moher D, Liberati A, Tetzlaff J, Altman DG, The PRISMA Group (2009). Preferred Reporting Items for Systematic Reviews and Meta-Analyses: The PRISMA Statement. PLoS Med 6(7): e1000097. doi:10.1371/journal.pmed1000097

For more information, visit: **www.prisma-statement.org**.

Page 2 of 2

1. The protocol is under review and it is not published yet, this is the submission ID [↑](#footnote-ref-2)
